# Multilayer perceptron modeling for social dysfunction prediction based on general health factors in an Iranian women sample

**DOI:** 10.3389/fpsyt.2023.1283095

**Published:** 2023-12-14

**Authors:** Sajjad Bagheri, Sarvenaz Taridashti, Hojjatollah Farahani, Peter Watson, Elham Rezvani

**Affiliations:** ^1^Clinical Psychology, Department of Psychology, Hakim-Toos Institute of Higher Education, Mashhad, Iran; ^2^Industrial and Organizational Psychology, Department of Psychology, Montclair State University, Montclair, NJ, United States; ^3^Department of Psychology, Tarbiat Modares University, Tehran, Iran; ^4^MRC Cognition and Brain Sciences Unit, University of Cambridge, Cambridge, United Kingdom

**Keywords:** multilayer perceptron, artificial neural network, social dysfunction, anxiety, depression, low-income women

## Abstract

In the year 2022, this research conducted an in-person study involving 780 single or widowed women, aged between 20 and 70, falling within the bottom three economic deciles and possessing varying levels of education. All participants held educational qualifications below a high school diploma and were beneficiaries of charitable financial support in Khorasan province, Iran. The study aimed to investigate the predictive factors of social dysfunction in this specific demographic. Data collection spanned a 12-month period throughout 2022, with participants completing the GHQ-28 questionnaire during their visits to the charity office. Clinical in-person interviews were also conducted to gather comprehensive data. Data analysis was carried out using IBM SPSS version 27. The research employed a Multilayer Perceptron (MLP) neural network model, considering an extensive set of input factors and covariates. These factors included cognitive functioning, anxiety, depression, age, and education levels. The MLP model exhibited robust performance, achieving high overall accuracy and sensitivity in identifying cases of high social dysfunction. The findings emphasized the significance of cognitive functioning, anxiety, and depression as pivotal predictors of social dysfunction within this specific demographic, while education and age displayed relatively lower importance. The normalized importance scores provided a relative measure of each covariate’s impact on the model’s predictions. These results furnish valuable insights for the development of targeted interventions and evidence-based policies aimed at addressing social dysfunction and promoting societal well-being among economically disadvantaged, single or widowed women. Notably, the research underscores the potential of MLP modeling in social science research and suggests avenues for further research and refinement to enhance the model’s predictive accuracy, particularly for cases of low social dysfunction.

## 1 Introduction

Psychological well-being is a complex and multi-faceted aspect of human health, with mental disorders exerting a profound and lasting impact on individuals. Recognizing and addressing these disorders effectively is of paramount importance. To this end, the choice of appropriate assessment tools is the initial step in identifying and understanding these problems. One such tool frequently employed by researchers is the questionnaire, tailored to specific methodologies and target groups (1).

Social functioning, a cornerstone of an individual’s overall well-being and daily life, encompasses the capacity to form and sustain relationships, adapt to social contexts, and communicate effectively (2, 3). Understanding the intricate domain of social functioning is indispensable. Effective adult functioning hinges on the ability to interpret others’ behaviors and respond appropriately, guided by widely accepted norms of social interaction (4). An individual’s social functioning is defined by their interactions with their surroundings and their capacity to carry out their role within such contexts (5). Social dysfunction, encompassing a broad spectrum of maladaptive behaviors and impaired interactions, has become a pressing concern in contemporary society. It hampers the functioning of individuals, communities, and societies, leading to a range of adverse consequences (6). This underscores the importance of understanding and addressing social dysfunction as it not only affects individual well-being but also has far-reaching implications for the broader social fabric.

The psychosis spectrum presents three vital components of social ability: social cognition, social interaction, and social functioning. These components are intertwined, with competency in one skill often fostering another. Social cognition, in particular, stands as a linchpin of successful interactions, enabling and reinforcing effective social functioning (7). Understanding of the factors that contribute to social dysfunction is crucial for policymakers, clinicians, and researchers seeking to address these challenges effectively. Research on social dysfunction has revealed its multifaceted nature, influenced by various biological, psychological, and sociocultural factors. Previous studies have highlighted the role of cognitive functioning in shaping social behavior and interactions. Cognitive deficits have been linked to impaired social adaptability and difficulties in understanding and interpreting social cues (8).

Emotional states, particularly anxiety and depression, have also garnered attention in the context of social dysfunction. Individuals experiencing anxiety may withdraw from social interactions due to fear or discomfort, leading to social dysfunction (9). Similarly, depression can contribute to social withdrawal and impaired social functioning, as individuals may struggle with motivation and engagement in social activities. Recognizing the influence of anxiety and depression in predicting social dysfunction is essential for devising interventions that address emotional well-being. A linkage between the presence of social dysfunction in depression and anxiety has long been noted (10, 11).

Major depressive disorder (MDD) is a prevalent psychiatric condition characterized by persistent sadness and a loss of interest or pleasure in daily activities (12). Notably, MDD often leads to profound and pervasive impairments in social functioning, defined as the capacity to fulfill standard social roles (13). Importantly, depression frequently results in the loss or disruption of significant social relationships. Another critical clinical feature of depression is the presence of somatic symptoms which can be interpreted as the clinical manifestation of psychological issues (14, 15). According to research, women who exhibit a greater extent of physical symptoms are more prone to being diagnosed with depression (16). Although women are known to report higher somatic symptoms, less is known regarding within-group disparities between women of varying education and age.

Depression is one of the most prevalent mental disorders in the 21st century, and it is related to low socioeconomic status (17), poor physical health (18), sleep problems (19), and a drastic suicide risk (20). Annually, an estimated 703,000 individuals commit suicide throughout the world. For every suicide, there are about 20 others who attempt suicide and several more who have serious suicidal ideation (21). Suicide can be prompted by a variety of risk factors such as poor socioeconomic status, financial hardship, and a low educational level (22, 23). It has been observed in older people that prior adverse experiences influence the management of future crises (24), while others argue that these can lead to depression (25), while still, others contend that older people, in particular, should be considered for suicide prevention programs owing to the numerous risk factors that relate to them (26). The gender paradox in suicide is long-established, with women having more suicidal thoughts and attempts than males (27, 28) with a prevalence of suicidal ideation in women. However, the intra-group variation in the prevalence of suicidal ideation in women has not been widely investigated. Furthermore, these social deficits are linked to an array of negative outcomes, including increased mortality rates (29, 30). This intricate web of connections between suicide, social dysfunction, and depression highlights the urgent need for comprehensive approaches to address these interrelated issues. By understanding these relationships, we can develop more effective strategies for suicide prevention and mental health support, especially among at-risk populations.

Although depression is the most prevalent mental disorder, as many as 20 million people were found to have anxiety disorders worldwide.^1^ It appears that elderly people have lower prevalence rates of diagnosed anxiety disorders than younger adults (31). However, researchers have discovered a wide range in the prevalence of anxiety disorders during a 12-month period among adults aged 55 and above, ranging from 7 to 12% (32–34). In addition, the prevalence of disorders differs among cultures and living conditions, as well as depending on study design and evaluation methods (35–37). The symptoms of anxiety are more prevalent in low socioeconomic groups (38). It is well recognized that there are differences in mental health across conventional SES variables including income, educational achievement, and other measures of social rank (39). Prins et al. hint of under researched processes in social epidemiology by demonstrating that the effects of class relations on anxiety go beyond those of SES (40). Hence, there are multiple factors related to SES that should be taken into account while investigating anxiety.

In addition to emotional and cognitive factors, sociodemographic characteristics such as education and age have been explored in relation to social dysfunction. Education plays a significant role in social integration and cohesion, with higher educational attainment often associated with improved social adaptability and engagement (41). Age can also influence social functioning, as individuals at different life stages may face distinct social challenges and opportunities (42). Acknowledging the impact of education and age on social dysfunction can inform targeted interventions tailored to specific age groups. Social dysfunction is a multifaceted phenomenon that affects various aspects of patients’ lives, including social interactions, everyday activities, and employment status (43). Vividly, social dysfunction is a multidimensional phenotype that is impacted by a range of socio-demographic factors, such as poor socioeconomic status, as well as psychological disorders/dysfunctions such as depression and anxiety. People’s social roles have been found to change with age and can be influenced by psychopathology (44). Social dysfunction is correlated with unemployment, disability and being single (45, 46).

Understanding the complexity of social dysfunction requires considering various factors beyond just emotional and cognitive aspects. Sociodemographic characteristics like education and age have emerged as significant contributors to the dynamics of social dysfunction. Research has shown that education plays a pivotal role in promoting social integration and cohesion, with higher educational attainment often linked to enhanced social adaptability and engagement (41). Additionally, age can exert a profound influence on social functioning, as individuals at different life stages encounter diverse social challenges and opportunities (47). Social dysfunction, a multifaceted phenomenon impacting various facets of patients’ lives, encompasses social interactions, everyday activities, and employment status (32). This multidimensional phenotype is further shaped by a range of socio-demographic factors, including poor socioeconomic status, as well as psychological disorders and dysfunctions like depression and anxiety. Moreover, people’s social roles undergo transformation with age and can be influenced by psychopathology (44). Notably, social dysfunction exhibits strong correlations with unemployment, disability, and being single, as evidenced by studies such as those conducted by Brown et al. (45) and Rizvi et al. (46).

Machine learning, and specifically Multilayer Perceptron (MLP) modeling, has shown promise in capturing complex relationships and nonlinear dependencies among variables. In social science research, MLP models have been used to predict various social and behavioral outcomes. For instance, Zheng et al. (48) employed an MLP model to predict mental health outcomes, while Lu et al. (49) used MLPs to identify risk factors for social problems. These studies highlight the potential of MLP modeling in social science research, particularly in predicting complex outcomes such as social dysfunction. In recent years, advances in data science and machine learning have opened new avenues for studying complex social phenomena. Among the innovative methodologies, MLP modeling has emerged as a powerful tool for predictive analytics in various domains, including social science research. This study aims to utilize an MLP model to explore the impact of covariates and hidden layers on predicting social dysfunction, offering valuable insights for targeted interventions and policy formulation. The incorporation of covariates into MLP modeling offers an opportunity to gain insights into the impact of various factors on social dysfunction. Covariates encompass independent variables that may influence an individual’s behavior and interactions within society. In the context of social dysfunction, covariates could include demographic information, cognitive functioning, emotional states, and socio-economic status, among others. Understanding the relative importance of these covariates can shed light on the underlying mechanisms driving social dysfunction.

Research studies have demonstrated the utility of MLPs in various social science domains, including predicting mental health outcomes (48) and identifying risk factors for social problems (49). MLPs offer the advantage of uncovering hidden patterns and nonlinear associations, allowing researchers to explore complex interactions among covariates and the dependent variable. By utilizing this modeling approach, researchers can potentially develop more accurate and nuanced models for social dysfunction prediction. However, while existing literature has explored the individual impacts of cognitive functioning, emotional states, education, and age on social dysfunction, limited research has investigated their combined influence within an MLP modeling framework. This study seeks to address this gap by employing an MLP model that incorporates hidden layers to capture intricate relationships among these covariates. By doing so, this research aims to provide a nuanced understanding of social dysfunction prediction and identify the most influential factors for targeted interventions and policy development.

In this context, the present study utilizes an MLP model to predict social dysfunction by incorporating a set of covariates, including education, cognitive functioning, anxiety, depression, and age. By analyzing the importance of these covariates in the context of social dysfunction prediction, this research aims to contribute to a deeper understanding of the multifaceted nature of social dysfunction and provide valuable insights for designing targeted interventions and evidence-based policies. In this study we utilized network analysis which assumes that symptoms of disorders trigger one another (50). In the field of psychopathology, a network approach enables the assessment of how specific behaviors or symptoms are interconnected with several other behaviors or symptoms (51). Implementing network analysis allows for investigating how symptoms of disorders link and become mutually reinforcing by analyzing symptoms as a network (52).

In light of the vulnerability experienced by women in low socioeconomic status and the prevalence of employment inequalities, this study was undertaken to address the imperative need for enhanced research and initiatives aimed at safeguarding and enhancing women’s mental well-being. The primary objective of this study was to assess disparities in Cognitive functioning, social dysfunction, Anxiety, depression Education levels and age within this demographic, shedding light on the pressing mental health concerns faced by women in low socioeconomic groups.

The primary objective of this study is to assess and analyze the disparities in cognitive functioning, social dysfunction, anxiety, depression, education levels, and age within a specific demographic of women in low socioeconomic groups. By employing Multilayer Perceptron (MLP) modeling and incorporating covariates, we aim to gain a deeper understanding of the multifaceted nature of social dysfunction, identify the most influential factors affecting it, and provide valuable insights for designing targeted interventions and evidence-based policies to enhance the mental well-being of women in low socioeconomic status.

## 2 Materials and methods

### 2.1 Study methodology

The multilayer perceptron (MLP) neural network function of IBM SPSS v27 was utilized in this study to minimize errors in prediction. The architecture of the neural network consisted of three layers: an input layer, a hidden layer that incorporated radially symmetric functions and unsupervised learning to define the hidden neurons, and an output layer with a categorical node. This node was used to calculate the weighted sum of the hidden layer outputs and determine the class index for the input pattern. The researchers experimented with different combinations of nodes in one or two hidden layers to build the model. Neural networks learn from potential correlations between independent (cause criteria) and dependent (effect criteria) variables to create a model, and are able to justify the outcomes by linking predicted values with observed actual values. Neural network systems are more effective in such applications than traditional computing systems that rely on a set of instructions to solve problems.

### 2.2 Data collection and variables

The study sample consisted of 780 single or widowed women between the ages of 20 and 70, who fell within the bottom three economic deciles and had varying levels of education. All participants had less than a high school diploma and were receiving charitable financial support in Khorasan province, Iran. Data were collected over a 12-month period during the winter to fall of 2022, with participants completing the questionnaire and clinical in-person interview during their visits to the charity office.

#### 2.2.1 Inclusion and exclusion criteria

##### 2.2.1.1 Inclusion criteria

Single or widowed women.

Aged between 20 and 70.

Falling within the bottom three economic deciles.

Having varying levels of education, all participants having less than a high school diploma.

Receiving charitable financial support in Khorasan province, Iran.

##### 2.2.1.2 Exclusion criteria

Any individuals not meeting the above inclusion criteria.

### 2.3 Ethical statement

Before administering the GHQ-28 questionnaire, the objective of the study was explained to all participants, and their consent was obtained. They were made aware of their right to withdraw from the study at any time without consequence. For those who had difficulty reading the questionnaire due to physical or academic limitations, an assistant read the questions and recorded their responses. This approach ensured that all participants had an equal opportunity to participate in the study, regardless of their literacy level or any other potential barriers to participation.

### 2.4 Measurements

#### 2.4.1 GHQ 28

The mental health status of the study participants was assessed using the General Health Questionnaire-28 (GHQ-28), a validated instrument developed by Goldberg and Hillier (53). This questionnaire is designed to comprehensively evaluate various aspects of mental health, consisting of four distinct sub-components: Somatic symptoms (items 1–7), Anxiety/insomnia (items 8–14), Social dysfunction (items 15–21), and Severe depression (items 22–28), each comprising seven items for a total of 28 items. Participants provided responses to these items using a Likert scale ranging from zero to three, with lower scores indicating better mental health. The GHQ-28 demonstrated strong internal consistency (Cronbach’s alpha coefficient of 0.9), good split-half reliability (coefficient of 0.89), and moderate test–retest reliability (coefficient of 0.58), indicating its stability over time (54). Factor analysis identified four distinct factors, namely “depression,” “psychosocial activity,” “anxiety,” and “somatic,” elucidating the multidimensional nature of the mental health assessment. Furthermore, the study population-specific receiver operating curve (ROC) analysis determined an optimum cutoff score of 19/20, with a sensitivity of 0.83 and specificity of 0.76, facilitating the identification of individuals at higher risk of mental health issues. This brief and practical assessment tool can be completed in approximately 5 min, making it an efficient choice for mental health evaluation in the context of the study.

### 2.5 The ANN approach

Artificial neural networks (ANNs) are computational methods that simulate animal brain processes in a simplified manner and are widely used to solve complex problems (55). These networks consist of artificial neurons or nodes that act as information processing units. These neurons are organized in layers and connected through synaptic weights or connections. Using this processing style, the neurons can screen and communicate data in a controlled manner to create an analytical model that can classify stored data. ANNs typically consist of three interconnected layers of artificial neurons: the input layer, the hidden layer, and the output layer. Researchers have the option to create one or more hidden layers between the input and output layers. Neurons within the same layer are not interconnected, but each neuron can be connected to a neuron in the next layer.

The first layer, known as the input layer, gathers data about variables from the provided dataset. The hidden layer then processes this data, and the output layer produces the categorical class label or predicts continuous measures. The values in the input layer that connect to the hidden node are multiplied by pre-determined weights, and then all of the results are summed to create a single number. This number is passed as an argument to a nonlinear mathematical function, called the activation function (AF) in ANNs (56). The AF returns an output between 0 and 1 which is the sum of the weighted input values that enter a node. The activation function converts the weighted input of the neuron to its output activation. Artificial neural networks (ANNs) neurons go through two stages: the training stage and the usage stage. During the training stage, the system is taught to predict outputs using datasets with actual inputs and outputs as examples. This supervised learning begins with random weights and adjusts the weights using gradient-based optimization algorithms like back-propagation to solve the problem. The difference between the target output values and the obtained values is calculated using the error function to regulate learning (57). The error function is dependent on the weights, which must be adjusted to minimize the error. After a dataset of respectable weights has been originated, the neural network model can take an alternative set with unidentified output measures and forecast the corresponding outputs automatically.

### 2.6 The multilayer perceptron approach

The perceptron-based model is only suitable for linearly identifiable data. In cases where the dataset is non-linear, the multilayer perceptron (MLP) is used instead. The MLP is a neural network with interconnected neuron layers that can incorporate the high non-linearity of the dataset. By using non-linear activation functions, such as the sigmoid function, the MLP is capable of approximating any continuous function at a random minor error by applying complex enough MLPs. The MLP network training procedure is used to minimize an objective function with regard to its criteria, which is connected to the task that the MLP is used for. The feedforward algorithm is used to quickly complete the prediction by an MLP (56).

The back-propagation algorithm (BPA) is widely used to change the ANN weights to lessen the mean squared error between the desired and actual outputs of the network. BPA uses controlled learning in which the neural network is trained using a dataset for which the input, as well as the desired outputs, are known. After the training process, the network weights are identified and then are used to compute the output measures for the original input samples. The gradient method can be used to reduce the objective function E(θ), which states that the sum of an update for a parameter is negatively proportionate to the gradient at its current value (56, 58–61).

#### 2.6.1 The number of the necessary hidden units

The number of hidden units in an MLP is crucial to achieve a desired approximation level and influences the number of independent values that need to be adjusted in the network parameters. However, determining the necessary number of MLP parameters is not straightforward, especially when the hidden units are distributed across different layers. It is important to find the optimal number of hidden units, and also to define the maximum number of parameters for a given number of hidden units. Typically, one hidden layer is sufficient, but in some cases, two hidden layers may be needed to meet the required number of network constraints. The necessary number of hidden units can be calculated using equations that take into account the input quantity and the desired degree of model fit. Equations are presented to help compute the number of required hidden units and their distribution across one or two hidden layers. The equations consider the number of inputs and the desired degree of fit, and can be used to determine the necessary number of parameters for MLP neural networks. Typically, one hidden layer is enough, but if two hidden layers are needed to meet the required constraints, the equations can help determine the appropriate number of hidden units for each layer (56, 62, 63).

### 2.7 Network specifications

The predictive model employed in this study is based on the Multilayer Perceptron (MLP) architecture. MLPs are a class of artificial neural networks consisting of interconnected layers of nodes, where each node performs a weighted sum of inputs and applies an activation function to produce an output. The model architecture includes an Input Layer, two Hidden Layers, and an Output Layer. The Input Layer contains 18 units, representing the covariates and input factors. The two Hidden Layers consist of 8 and 6 units, respectively, and are activated using the hyperbolic tangent activation function. The Output Layer comprises 2 units, representing the binary classification of social dysfunction, and uses the identity activation function. Specifications of MLP artificial neural network have been presented in Table 1.

**Table 1:** Specifications of MLP artificial neural networks used in this research.

Metric	Training phase	Testing phase
Sum of squares error	45.444	24.565
Incorrect prediction rate (%)	11.1	11.9
Accuracy (%)	-	88.9
Sensitivity (high dysfunction)	-	97.7
Sensitivity (low dysfunction)	-	34.2

### 2.8 Data preprocessing and rescaling

Before feeding the data into the MLP model, standardization was applied to the covariates to ensure a consistent scale and facilitate convergence during training. Standardization centers the data around zero with a standard deviation of one, eliminating potential issues related to varying scales across variables. This step ensures that each covariate contributes equally to the model’s predictions and prevents any particular covariate from dominating the learning process (56).

### 2.9 Model training and error function

Farahani et al. (56) describe that the MLP model was trained using a supervised learning approach, where the model is presented with a labeled dataset and iteratively adjusts its weights and biases to minimize prediction errors. The error function used for training is the Sum of Squares, which calculates the squared difference between the predicted output and the true target value for each data point. The backpropagation algorithm, a common method for training MLPs, is employed to update the model’s parameters during each iteration, optimizing the model’s performance over time.

### 2.10 Model evaluation and testing

To evaluate the performance of the MLP model, it was tested on an independent dataset that was not used during training. The testing sample allowed us to assess the model’s generalization ability and its ability to make accurate predictions on unseen data. The model’s performance was assessed using metrics such as Sum of Squares Error and Percent Incorrect Predictions. Additionally, the classification performance of the model was evaluated using metrics such as sensitivity and specificity to assess its ability to correctly identify cases of high and low social dysfunction.

## 3 Results

The results of our study utilizing the Multilayer Perceptron (MLP) model demonstrated strong predictive performance in assessing social dysfunction. The demographic characteristics of our study sample are summarized in Table 2, indicating key features of the participants, including their educational levels and age categories. A total of 780 participants were included in the analysis (Table 3).

**Table 2:** Normalized importance of covariates.

Covariate	Normalized importance (%)
Cognitive (HYPO)	95.2
Anxiety (ANX)	100.0
Depression (DEP)	97.2
Depression (DEP)	97.2
Education (EDU)	37.7
Age	24.1

**Table 3:** Demographic characteristics of the study sample.

Characteristic	Frequency	Percent
Education		
1 = Primary School	224	28.7
2 = Secondary School	230	29.5
3 = High School	326	41.8
Age		
1 = Over 35 years old	438	56.2
2 = Under 35 years old	342	43.8

The Multilayer Perceptron (MLP) model demonstrated promising performance in predicting social dysfunction based on the input factors and covariates. The model was trained and tested on a sample of data, with the training phase yielding a Sum of Squares Error of 45.444 and an 11.1% rate of incorrect predictions. During the testing phase, the model achieved a Sum of Squares Error of 24.565 and an 11.9% rate of incorrect predictions, indicating its ability to generalize well to unseen data.

The classification performance of the model was evaluated using the testing sample. The results revealed a high overall accuracy of 88.9%. The model exhibited a remarkable ability to correctly predict cases of high social dysfunction, with a sensitivity of 97.7%. However, its performance in identifying cases of low social dysfunction was relatively lower, with a sensitivity of 34.2%. These findings suggest that the MLP model is particularly adept at identifying individuals with high social dysfunction, but further improvements may be necessary to enhance its predictive accuracy for low social dysfunction cases.

An essential aspect of our study was assessing the relative importance of each covariate in predicting social dysfunction. We quantified the importance of cognitive functioning (HYPO), which emerged as the most influential factor, with an importance score of 0.269. Anxiety (ANX) closely followed, with a score of 0.282, indicating its strong predictive value. Depression (DEP) exhibited high predictive importance as well, with a score of 0.274. In comparison, education (EDU) and age played relatively smaller roles, with importance scores of 0.107 and 0.068, respectively. These importance scores provide a comparative measure within the model, indicating the relative impact of each covariate on social dysfunction prediction. The prominence of cognitive functioning and emotional states underscores the imperative for targeted interventions and support in these domains (Table 4).

**Table 4:** Importance of covariates in predicting social dysfunction.

Rank	Covariate	Importance score
1	Cognitive (HYPO)	0.269
2	Anxiety (ANX)	0.282
3	Depression (DEP)	0.274
4	Education (EDU)	0.107
5	Age	0.068

Finally, the study assessed the normalized importance of each covariate, offering insights into their relative contributions to the model’s accuracy. Although the contribution of depression (DEP) is higher (97.2%) than that of cognitive functioning (HYPO; 95.2%), it’s important to understand that the normalized importance score does not directly translate to absolute influence. The 100% contribution from anxiety (ANX) underscores its critical role in predicting social dysfunction, but this does not necessarily mean it is inherently more influential than other variables. The normalized importance scores for education (EDU) and age, at 37.7 and 24.1%, respectively, indicate that these factors have a comparatively lower impact on the model’s predictions when compared to other variables (Table 2).

**Figure 1:**
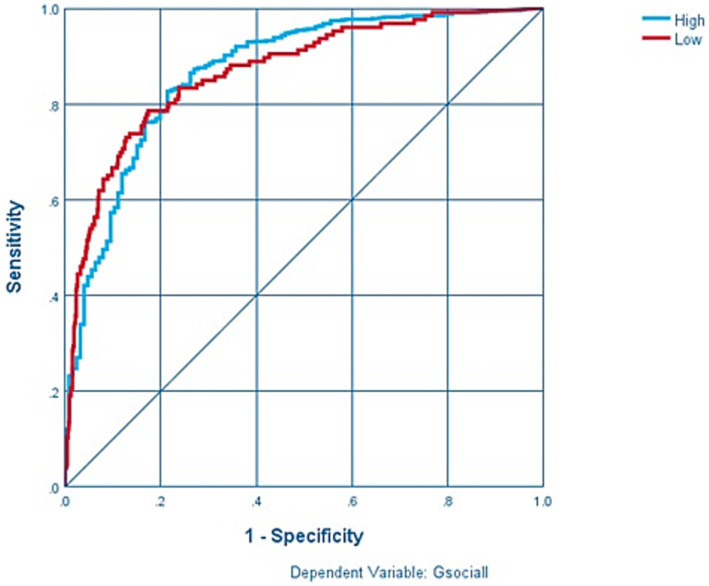
Sensitivity and specificity in MLP model.

**Figure 2:**
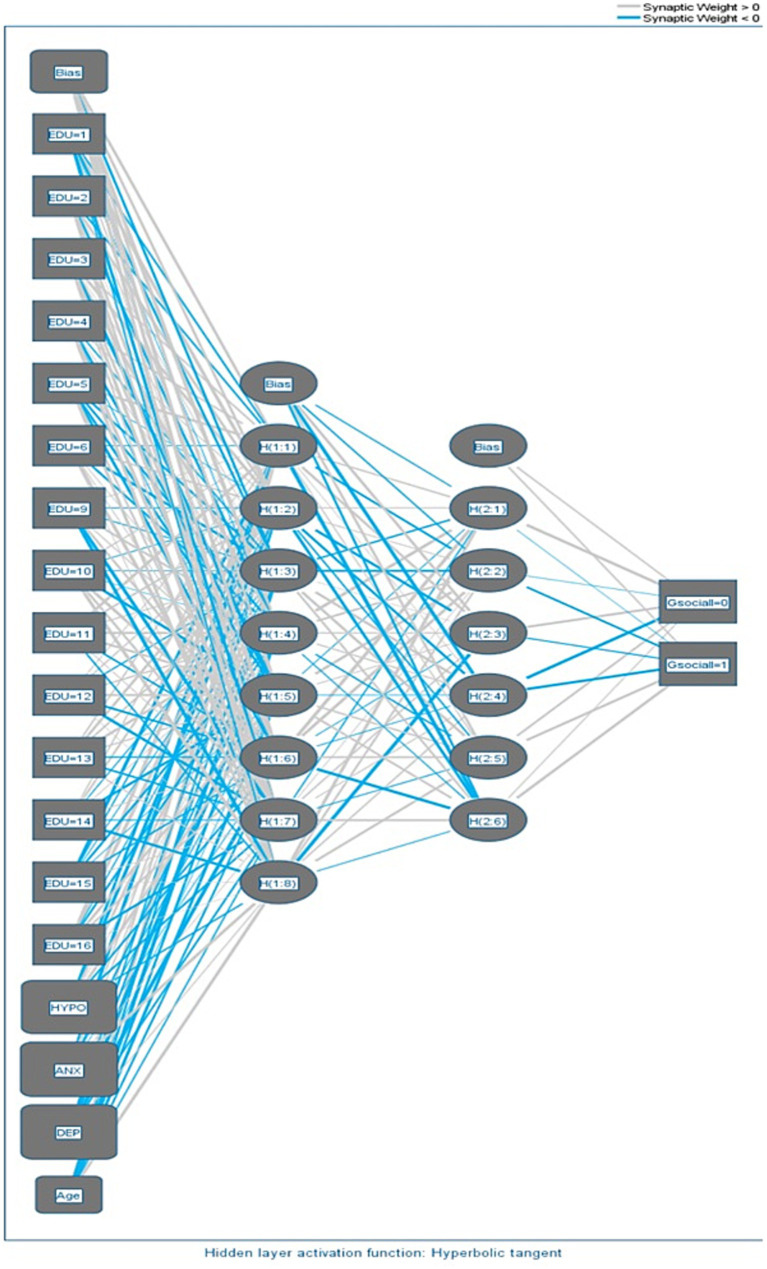
Network diagram.

The results of the Multilayer Perceptron (MLP) model for social dysfunction prediction are promising, demonstrating its capacity to capture complex relationships and dependencies among covariates. The model showcased high overall accuracy and strong sensitivity in identifying individuals with high social dysfunction. Cognitive functioning (HYPO), depression (DEP) and anxiety (ANX) emerged as the most influential predictors, highlighting their significance in predicting social dysfunction. These findings offer valuable insights for developing targeted interventions and evidence-based policies to address social dysfunction and promote societal well-being (Figures 1, 2).

To provide a more comprehensive understanding of the implications of our findings, it’s essential to discuss how the model’s performance aligns with our research objectives. Our study aimed to predict social dysfunction among a specific demographic of women in low socioeconomic status, and the results show the MLP model’s promising ability to achieve this goal. The model’s high overall accuracy and sensitivity are encouraging, as they indicate that it excels in identifying individuals with high social dysfunction. These findings hold significant promise for practical applications and policy development. By recognizing that cognitive functioning (HYPO), anxiety (ANX), and depression (DEP) are the most influential factors in predicting social dysfunction, we can now tailor interventions and policies to address these critical areas. For instance, targeted support programs for improving cognitive functioning, mental health, and coping with anxiety may be developed to aid individuals experiencing social dysfunction. Policymakers can use these insights to prioritize resources and design evidence-based initiatives. While our MLP model excels in identifying high social dysfunction cases, it’s important to acknowledge that further improvements are needed to enhance its predictive accuracy for low social dysfunction cases. Understanding the challenges and limitations of the model’s performance can guide future research and model refinement efforts. Future studies may focus on refining the model to better capture nuanced variations in social dysfunction among individuals with relatively lower dysfunction levels.

## 4 Discussion

The findings derived from the Multilayer Perceptron (MLP) model, employed to forecast social dysfunction, provide valuable insights into the intricate nuances of this societal concern. The model’s remarkable performance, characterized by its elevated overall accuracy and sensitivity in identifying instances of high social dysfunction, underscores its efficacy in discerning the intricate interplay among input variables and covariates. Nevertheless, the comparatively lower sensitivity in detecting cases of low social dysfunction highlights the imperative for further exploration and enhancement of the model’s predictive capabilities in this particular domain. One of the remarkable attributes of artificial neural networks is their adaptability, achieved through the fine-tuning of model factors via weight adjustments, allowing for specific function training, as elucidated by Hagan and Dennutu (64). Furthermore, the inherent parallel processing capabilities of artificial neural networks translate into swift classification speeds, as articulated by the same authors. Additionally, they help mitigate the oversimplification of complex relationships, a notable advantage. These merits underscore the preference for employing this method over traditional statistical techniques such as logistic regression for predictive purposes.

Our study findings, echoing the insights of Farahani et al. (65), underscore the significant role of artificial neural networks in making precise predictions based on various risk factors. Farahani et al.’s (65) research, which achieved an impressive 90.5% accuracy in classifying high and low-risk individuals in predicting suicide tendencies, highlights the potential of these networks in modeling complex, non-linear relationships. In essence, both of these research work underline the potential of artificial neural networks in unraveling complex social phenomena and stress the importance of addressing mental health concerns in interventions for healthier and thriving communities.

The finding that cognitive functioning (HYPO) emerged as an influential predictor of social dysfunction aligns with existing literature on the role of cognitive processes in shaping human behavior and interactions (66). Cognitive functioning encompasses a broad range of mental processes, such as memory, attention, and problem-solving, which play a pivotal role in social functioning and adaptability. The strong impact of cognitive functioning on social dysfunction underscores the significance of cognitive assessments and interventions in mitigating social challenges.

Anxiety and depression were also identified as crucial predictors of social dysfunction. These findings are consistent with research that highlights the association between mental health issues and impaired social interactions (67). Individuals experiencing anxiety or depression may struggle with social interactions, leading to withdrawal and social dysfunction. The MLP model’s ability to capture the influence of these emotional states emphasizes the importance of addressing mental health concerns in interventions aimed at promoting healthy social functioning (45).

Education and age have demonstrated comparatively lower predictive significance in relation to social dysfunction when juxtaposed with cognitive functioning, anxiety, and depression. Nevertheless, their roles remain substantively meaningful and warrant careful consideration in comprehensive analyses. Education, for instance, has been consistently correlated with heightened social integration and cohesion, often highlighting that individuals with higher educational attainment tend to exhibit superior social adaptability (41). Similarly, age can exert a notable influence on social functioning, with individuals at various life stages grappling with distinct social challenges (47). Recognizing the pivotal roles of education and age in the context of predicting social dysfunction paves the way for tailoring age-appropriate interventions and support systems.

The relative impact of each covariate on the model’s predictive accuracy is further underscored by the normalized importance scores. Notably, cognitive functioning and depression exhibit substantial normalized importance, signifying their significant contributions to the model’s overall performance. Furthermore, the comprehensive normalized importance attributed to anxiety underscores its pivotal role in precisely predicting social dysfunction. This understanding of the varying importance of covariates enables the prioritization and customization of interventions to effectively target the most influential factors. The current study serves as a compelling testament to the potential of Multilayer Perceptron (MLP) modeling in unraveling intricate social phenomena, such as social dysfunction. The model’s capacity to harness hidden layers and capture nonlinear relationships among covariates offers an invaluable tool for deciphering the multifaceted dynamics that underlie social dysfunction. As MLP modeling continues to evolve, it opens up new avenues for the development of refined predictive models and a nuanced comprehension of social behavior, thereby supporting the formulation of evidence-based policies and the implementation of precisely targeted interventions.

In summation, the discussion of the MLP model’s outcomes underscores the critical significance of cognitive functioning, anxiety, and depression as major predictors of social dysfunction. These findings accentuate the imperative of addressing mental health concerns and cognitive well-being within interventions aimed at fostering healthy social interactions. Moreover, the acknowledgment of the roles played by education and age provides insights for tailoring interventions tailored to specific age groups and educational backgrounds. The application of MLP modeling in social science research presents a promising avenue toward gaining deeper insights into social dysfunction and the formulation of effective strategies to nurture thriving communities.

### 4.1 Limitations

1. Sample size and generalizability: one of the primary limitations of this study is the sample size. While the research provides valuable insights into social dysfunction among a specific group of single or widowed women in Iran, the relatively small sample size may limit the generalizability of the findings. Future studies could benefit from larger and more diverse samples, encompassing various demographic groups.
2. Data collection bias: the study relied on self-report data and in-person interviews, which may introduce response and recall biases. Participants might underreport or overreport certain aspects of their experiences due to social desirability or memory limitations. To mitigate this bias, incorporating additional data collection methods, such as observations or ecological momentary assessments, could enhance the accuracy of the findings.
3. Single gender and age group: the study’s focus on single or widowed women in Iran within a specific age range might limit the applicability of the findings to a broader population. Future research should aim for more diverse samples to ensure the results can be applied to a wider range of individuals.
4. Neural network complexity: while the MLP model proved effective, it is essential to acknowledge that the model’s complexity can be a double-edged sword. Overfitting, where the model performs exceptionally well on the training data but struggles with unseen data, can be a concern. Careful consideration of the model’s architecture and ongoing validation with larger datasets can help address this concern.

### 4.2 Suggestions for future research

1. Diverse and representative samples: future research should endeavor to include more diverse and representative samples, both in terms of demographics and geographical locations. This will allow for a more comprehensive understanding of social dysfunction across different populations.
2. Longitudinal studies: longitudinal studies tracking participants over an extended period can provide a deeper understanding of how social dysfunction evolves over time and the factors that contribute to its persistence or resolution.
3. Incorporate objective measures: combining self-report data with objective measures can help mitigate response bias and provide a more accurate representation of individuals’ experiences. For example, using wearable technology to collect real-time data on social interactions can enhance the quality of data.
4. Comparative analyses: conducting comparative analyses between different machine learning models and traditional statistical approaches, such as logistic regression, can offer insights into the strengths and weaknesses of each method. This can help identify which approach is most suitable for specific research questions.
5. Intervention development: building on the insights regarding cognitive functioning, anxiety, depression, and other influential factors, future research can focus on developing and testing targeted interventions to mitigate social dysfunction. These interventions should consider a variety of factors, including cognitive well-being, mental health, education, and age, to promote healthy social interactions.
6. Validation and external testing: it is essential to validate the findings in different cultural and geographical contexts. External testing can confirm the robustness and applicability of the MLP model for predicting social dysfunction in diverse settings.
7. Advanced data collection techniques: leveraging advanced data collection techniques, such as natural language processing for text data or physiological monitoring for stress-related data, can provide a more comprehensive picture of social dysfunction’s underlying mechanisms.

## 5 Conclusion

The present study utilized a Multilayer Perceptron (MLP) model to predict social dysfunction, drawing insights from a comprehensive set of input factors and covariates. The MLP model demonstrated favorable performance in both training and testing phases, highlighting its capability to generalize well to unseen data and make accurate predictions. The high overall accuracy of 88.9% underscores the effectiveness of the MLP model in classifying social dysfunction cases.

An essential aspect of the study was the assessment of covariate importance in predicting social dysfunction. Cognitive functioning (HYPO) emerged as an influential predictor, emphasizing its critical role in shaping social behavior and interactions. Anxiety (ANX) and depression (DEP) also contributed significantly to the model’s accuracy. These findings offer valuable insights into the drivers of social dysfunction and call for targeted interventions addressing cognitive functioning and emotional well-being.

The normalized importance scores provided further clarity on the relative contributions of covariates to the model’s predictive accuracy. Anxiety (ANX), cognitive functioning (HYPO) and depression (DEP) demonstrated the highest relative importance, indicating their significant impact on the model’s performance. Anxiety (ANX), in particular, exhibited a full contribution, indicating its indispensable role in predicting social dysfunction. Education (EDU) and age, though less influential, remain important factors in understanding social dysfunction dynamics.

Overall, the study highlights the potential of MLP modeling in social science research, particularly in predicting social dysfunction. The model’s ability to capture complex relationships and nonlinear dependencies among covariates makes it a valuable tool for unraveling the intricate mechanisms underlying social dysfunctions. The findings presented here contribute to a deeper understanding of social dysfunction’s multifaceted nature and inform evidence-based interventions and policies aimed at addressing these challenges.

While the MLP model showed robust performance, there is room for further research and refinement. Enhancing the model’s sensitivity in identifying low social dysfunction cases could be explored to improve its comprehensive predictive capacity. Additionally, expanding the dataset and incorporating additional relevant covariates may enrich the model’s predictive power, providing a more nuanced understanding of social dysfunction dynamics.

In conclusion, the study underscores the significance of Multilayer Perceptron Modeling in social dysfunction prediction, offering valuable insights into the impact of covariates on this complex societal issue. The results have implications for policymakers, clinicians, and researchers seeking to address social dysfunction and promote healthier and more harmonious communities.

## Data availability statement

The raw data supporting the conclusions of this article will be made available by the authors, without undue reservation.

## Ethics statement

The studies involving humans were approved by Tarbiat Modares University, Tehran, Iran. The studies were conducted in accordance with the local legislation and institutional requirements. The participants provided their written informed consent to participate in this study. Written informed consent was obtained from the individual(s) for the publication of any potentially identifiable images or data included in this article.

## Author contributions

SB: Writing – original draft. ST: Writing – original draft. HF: Methodology, Writing – original draft, Writing – review & editing. PW: Methodology, Writing – review & editing. ER: Writing – original draft.

## Footnotes

^1^https://ourworldindata.org/mental-health
